# Structure, Stability, and Superconductivity of Two-Dimensional Janus NbSH Monolayers: A First-Principle Investigation

**DOI:** 10.3390/molecules28145522

**Published:** 2023-07-19

**Authors:** Yan Li, Chunying Pu, Dawei Zhou

**Affiliations:** 1 International Joint Research Laboratory of New Energy Materials and Devices of Henan Province, Institute for Computational Materials Science, School of Physics and Electronics, Henan University, Kaifeng 475004, China; lydyx730@163.com; 2 Henan International Joint Laboratory of MXene Materials Microstructure, College of Physics and Electronic Engineering, Nanyang Normal University, Nanyang 473061, China

**Keywords:** two-dimensional materials, first-principles calculations, structure prediction, Janus materials

## Abstract

Two-dimensional Janus materials have unique structural characteristics due to their lack of out-of-plane mirror symmetry, resulting in many excellent physical and chemical properties. Using first-principle calculations, we performed a detailed investigation of the possible stable structures and properties of two-dimensional Janus NbSH. We found that both Janus 1T and 2H structures are semiconductors, unlike their metallic counterparts MoSH. Furthermore, we predicted a new stable NbSH monolayer using a particle swarm optimization method combined with first-principle calculations. Interestingly, the out-of-plane mirror symmetry is preserved in this newly found 2D structure. Furthermore, the newly found NbSH is metallic and exhibits intrinsic superconducting behavior. The superconducting critical temperature is about 6.1 K under normal conditions, which is found to be very sensitive to stress. Even under a small compressive strain of 1.08%, the superconducting critical temperature increases to 9.3 K. In addition, the superconductivity was found to mainly originate from Nb atomic vibrations. Our results show the diversity of structures and properties of the two-dimensional Janus transition metal sulfhydrate materials and provide some guidelines for further investigations.

## 1. Introduction

Since graphene was experimentally synthesized in 2004 [1], two-dimensional (2D) materials have attracted increasing attention of scientists, due to their excellent physical and chemical properties. From binary to ternary, a large number of two-dimensional materials have been discovered and synthesized [2], showing unexpected physical and chemical properties [3,4,5,6,7,8]. Unlike other 2D materials, Janus 2D materials are unique 2D materials with an asymmetric structure. This particular type of 2D Janus materials, with different properties on its two sides, cannot commonly be found in nature. The concept of 2D Janus materials was initially theoretically proposed in 2011 for studying the hydrogenation and halogenation of graphene and its derivatives [9]. It was not until 2013 that Zhang et al. successfully synthesized the first real Janus graphene in a laboratory setting, confirming its existence [10]. Since then, many 2D Janus transition metal (TM) dichalcogenides (JTMDCs) like MoSSe [11,12], WSSe thin-film [11], and Janus PtSSe [13] were successfully synthesized. Structurally, one side of JTMDs is composed of one type of transition metal dichalcogenide, while the other side is formed by another different type of transition metal dichalcogenide, hence the out-of-plane structural symmetry is broken [14]. 2D Janus MoSSe/MoSeS, for example, can be synthesized by selectively replacing the top layer chalcogen atom of MoS_2_/MoSe_2_ with other chalcogen (Se/S) atoms [12,15]. The novel structural characteristics with the breaking out-plane symmetry make 2D Janus materials have great potential applications in many special fields.

For example, the JMTDs have been found to be excellent catalyst materials. For example, the hydrogen evolution reaction (HER), the practical application of monolayer transition metal dichalcogenides (TMDs) as catalysts, has faced limitations due to the scarce number of catalytically active sites, while the Janus monolayer MoXY (X/Y = S, Se, and Te) materials exhibit exceptional catalytic performances due to the presence of edge sites [16]. It was found that both the Mo-edge and chalcogen atomic edges of Janus monolayer MoXY exhibit catalytic activity, resulting in a better catalytic performance of MoXY compared to that of monolayer MoS_2_. Furthermore, both the monolayer and multilayer of Janus MoSSe have been identified as efficient photocatalysts for water splitting [17]. These materials show excellent capability in harvesting solar energy and converting it into hydrogen fuel. In addition, the introduction of doping As and Si atoms in the S or Se sites of the 2D JTMDs VSSe monolayer greatly enhances HER performance [18]. This type of doping results in a significant reduction of the hydrogen adsorption free energy, which is even better than that of the traditional Pt catalyst. The Janus Pd-based TMD monolayers, including PdSSe, PdSTe, and PdSeTe, have also been found to have the ability to simultaneously facilitate the hydrogen and oxygen evolution reactions as efficient photocatalysts for water splitting [19].

The breaking of out-plane symmetry in Janus 2D materials allows the creation of special heterogeneous junctions with unexpected physical and chemical properties. For instance, a van der Waals heterostructure consisting of Janus MoSSe and boron pnictide (BP, BAs) monolayers has shown tremendous potential in various fields, such as nanoelectronics, optoelectronics, piezotronics, photovoltaics, low-power digital data processing, and memory devices [20]. JTMDs also demonstrate their superiority as gas sensing materials. In particular, the Janus MoSSe are found to be a superior gas sensing material with higher gas sensitivity, surface, and strain selectivity [21]. Furthermore, enhanced piezoelectric characteristics of 2D Janus materials compared to their pristine structures have been observed [22,23]. Several 2D Janus materials have been predicted theoretically to exhibit out-of-plane piezoelectricity, including MoSSe [24], M_2_XY (M = Ga, In, and X, Y = S, Se, Te) [23], and multilayer MoSTe [24]. For MoSSe, the out-of-plane piezoelectricity has been finally identified experimentally [12]. The 2D Janus MoSTe [25], ZnAXY [26] (A = Si, Ge, Sn, and X/Y = S, Se, Te, X < Y), and BMX_2_ (M = Ga, In, and X = S, Se) [27] monolayers were also predicted to have excellent electronic, spintronic, and piezoelectric properties. In addition to piezoelectricity, 2D Janus monolayers possess other interesting properties such as magnetism [28], valley polarization [29,30], and Rashba spin splitting (RSS) [31,32]. The RSS effect refers to the momentum-dependent spin splitting in bands that occurs due to spin–orbit coupling (SOC) in asymmetric structures. Hence the Janus 2D materials, with their natural antisymmetric structure, exhibit RSS in the presence of SOC. This property allows the control of electron current by manipulating spin precession, which forms the basis of spin field-effect transistors (SFETs) [33] and spin injectors [34]. Recent studies have shown that the strong SOC and mirror asymmetry of 2D Janus materials give rise to the RSS phenomenon, making them promising candidates for next-generation spintronic devices. For example, the Janus Ge_2_XY (X ≠ Y = P, As, Sb, and Bi) monolayers exhibit the out-of-plane piezoelectricity and giant Rashba spin-band splitting [35]. The Janus 2D materials Bi_2_X_3_ (X = S, Se) monolayers were reported to exhibit coexistence of anisotropic colossal out-of-plane piezoelectricity, giant RSS, and ultrahigh carrier mobilities [36]. The Janus Sn_2_XY (X/Y = S, Se, Te) monolayers were also found to have strong piezoelectricity with high electron mobility [37]. All the reported 2D Janus materials show great potential for nano-electronic applications.

Very recently, a Janus 2H-MoSH monolayer was synthesized through the selective removal of the top-layer sulfur atoms with hydrogen atoms [12]. The 2D Janus 2H-MoSH exhibits a metallic nature, and was predicted to be a 2D superconductor [38]. Recently, the 2H/1T-MoSH monolayers were investigated theoretically by Wang [39]. In their work, the Janus 1T-MoSH was found to be a charge-density wave (CDW) material, and both 1T and 2H-MoSH possess a superconducting state, with the superconducting behavior able to change greatly when they are subjected to strain.

Two-dimensional TMDS form a large family, and have different phases such as 2H, 1T, 1T′, and 2a × 2a phases etc. [40]. The subsequent 2D transition metal sulfhydrate materials (TM-SH) might also have different phases, so further investigation of the other TM-SH might bring us newer 2D materials with exciting properties. Among the various metal transition metal dichalcogenides (TMDCs), 2H-NbS_2_ stands out as a unique case. In contrast to other TMDCs such as 2H-NbSe_2_ and 2H-MoS_2_, 2H-NbS_2_ is the only known superconductor without any charge density wave (CDW) instabilities [41]. This observation is quite unusual and suggests a different behavior of 2H-NbS_2_ compared to its isostructural and isoelectronic counterparts. Based on this understanding, we anticipate that Janus NbSH will exhibit significant differences in both structure and properties compared to Janus MoSH. Considering the contrasting behavior of 2H-NbS_2_ and 2H-MoS_2_, it is expected that Janus NbSH may exhibit distinct properties not observed in Janus MoSH. These different features could manifest themselves in various aspects, including electronic, structure, and stability, making Janus NbSH an intriguing material for further exploration and potential applications.

In this paper, we examined already known phases of NbSH monolayers such as 2H and 1T, and also searched the possible stable phases using the swarm method together with first-principle calculations. The structure prediction method can search the potential energy surface of NbSH more widely and find more stable phases. This is especially true for the 2D Janus structure, which might exist as a metastable state because all the same type of atoms are on the same side. Just as we thought, a new stable NbSH phase with the out-of-plane mirror symmetry was first identified. We then investigated the properties of three NbSH phases such as structural stabilities, electronic structures, mechanical properties, and superconducting behavior. We believe that the investigations of the structural properties for 2D NbSH phases in this work could provide some important clues for further theoretical and experimental investigations of the 2D TM-SH materials.

## 2. Computational Methods

The global minimum structures of two-dimensional NbSH were predicted by using a first-principles swarm-intelligence structure search method implemented in the CALYPSO software [42,43]. Simulation cells containing 1, 2, and 4 formula units of NbSH were considered for structure-searching. Both the population size and the number of generations were set to 30 to ensure convergence. The calculations of the electronic and mechanical properties of NbSH were performed with the VASP package [44]. The projector augmented wave (PAW) method was adopted to analyze the interaction between the valence electron and the core ion, and the generalized gradient approximation in the form of the Perdew–Burke–Ernzerhof (PBE) [45,46] was used to estimate the exchange-correlation energy. The plane-wave cut-off energy was 520 eV, and the structure was relaxed until the residual force was less than 0.001 eVÅ^−1^/atom. The Brillouin zone was sampled using a k-point grid spacing of 2π × 0.03 Å^−1^ [47]. The vacuum thickness was set to 20 Å for eliminating interlayer interaction. The vdW interactions were treated using the (Grimme) DFT-D2 approximation [48]. Ab initio molecular dynamics simulations (AIMD) with the Nosé–Hoover thermostat and NVT ensemble [49] were used to estimate the thermal stability by using the 4 × 4 supercell at 300 K with the time step of 2 fs.

The superconducting transition temperature (*T*_C_) was calculated using the Allen–Dynes formula based on BCS theory [50]

(1) $$T_{C}=\frac{\omega_{\log}}{1.20}\exp(-\frac{1.04\;(1\;+\;\lambda )}{\lambda -\mu *(1\;+\;0.62\lambda )})$$

where $\omega_{\log}$, λ, μ* are the logarithmic average of the phonon energy, electron-phonon coupling constant, and the electron-electron coulomb repulsion parameter, respectively. The $\omega_{\log}$ and λ were calculated according to the two following formulas [51]:

(2) $$\omega_{\log}=\exp(\frac{2}{\lambda }\int_{0}^{\infty }\alpha^{2}F(\omega )\log\omega \frac{d\omega }{\omega })$$

(3) $$\lambda =2\int_{0}^{\infty }\frac{\alpha^{2}F(\omega )}{\omega }d\omega$$

where $\alpha^{2}F(\omega )$ is the electron-phonon spectral function, which can be further calculated in terms of the phonon linewidth as

(4) $$\alpha^{2}F(\omega )=\frac{1}{2\pi N(\varepsilon_{F})}\sum_{q\nu }\delta (\omega -\omega_{q\nu })\frac{\gamma_{q\nu }}{\hbar \omega_{q\nu }}$$

where $N(\varepsilon_{F})$, $\gamma_{q\nu }$ are the DOS at the Fermi level, and the linewidth of phonon mode at the wave vector q, respectively.

In this paper, λ and *T*c were calculated using the density functional perturbation theory as implemented in the QUANTUM ESPRESSO codes [52]. Ultrasoft pseudopotentials [53] for Nb, S, and H atoms were used, with a kinetic cutoff energy of 45 Ry. To obtain accurate values, the electron and phonon grids were set to 24 × 24 × 1 and 12 × 12 × 1, respectively. A Gaussian of width 0.02 Ry was used in the EPC calculations.

Mechanical properties such as elastic constant, Young’s modulus, and Poisson’s ratio were calculated using the VASPKIT software through an energy-strain method [54].

For two-dimensional materials, when only the x-y plane of the crystal is taken into account, the relationship between strain and stress can be expressed in a specific form as follows [55]

(5) $$\left(\begin{array}{c} \sigma_{1}\\ \sigma_{2}\\ \sigma_{3} \end{array}\right)=\left(\begin{array}{ccc} C_{11} & C_{12} & C_{16}\\ C_{21} & C_{22} & C_{26}\\ C_{61} & C_{62} & C_{66} \end{array}\right)\cdot \left(\begin{array}{c} \varepsilon_{1}\\ \varepsilon_{2}\\ \varepsilon_{6} \end{array}\right)$$

where *C*_ij_ (i, j = 1, 2, 6) is the in-plane stiffness tensor. The corresponding strain tensor $\varepsilon^{2D}$ can be expressed as follows, when a small strain ε is applied in a 2D crystal.

(6) $$\varepsilon^{2D}=\left(\begin{array}{ccc} \varepsilon_{1} & \varepsilon_{6}/2 & 0\\ \varepsilon_{6}/2 & \varepsilon_{2} & 0\\ 0 & 0 & 0 \end{array}\right)$$

Using the strain-energy method, the elastic strain energy per unit area can be expressed as follows [56]

(7) $$\Delta E(S,\{\varepsilon_{i}\})=\frac{S_{0}}{2}(C_{11}\varepsilon_{1}^{2}+C_{22}\varepsilon_{2}^{2}+2C_{12}\varepsilon_{1}\varepsilon_{2}+2C_{16}\varepsilon_{1}\varepsilon_{6}+2C_{26}\varepsilon_{2}\varepsilon_{6}+C_{66}\varepsilon_{6}^{2})$$

where $S_{0}$ is the equilibrium area of the system. $C_{\mathrm{ij}}$ is equal to the second partial derivative of the strain energy *E* with respect to strain ε, namely,

(8) Cij=1S0(∂2E∂εi∂εj)

As a consequence, for 2D materials, the unit of elastic constants is force per unit length (N/m) instead of GPa. There are six independent elastic constants for 2D materials: *C*_11_, *C*_12_, *C*_16_, *C*_22_, *C*_26_, and *C*_66_.

All three NbSH phases investigated in this paper crystalize in hexagonal structure; for the 2D hexagonal structure, there are only two independent elastic constants, *C*_11_ and *C*_12_. The elastic constants *C*_11_ and *C*_12_ were calculated via two strain modes as listed in Table 1. Finally, to obtain accurate elastic constants, a total of nine strains were used to fit the strain-energy Equation (7).

After obtaining the results for elastic constants *C*_ij_, we further evaluated angular-dependent Young’s modulus $Y_{2D}$ and Poisson’s ratio ν, to examine the mechanical characteristics of the three 2D structures. The angular-dependent $Y_{2D}(\theta )$ and ν(θ) were calculated with the following equations [57,58]

(9) $$Y_{2D}(\theta )=\frac{C_{11}C_{22}-C_{12}^{2}}{A^{4}C_{11}+B^{4}C_{22}-A^{2}B^{2}(2C_{12}-\prod )}$$

(10) $$\nu (\theta )=\frac{\left(A^{4}+B^{4})C_{12}-A^{2}B^{2}(C_{11}+C_{22}-\prod \right)}{A^{4}C_{11}+B^{4}C_{22}-A^{2}B^{2}(2C_{12}-\prod )}$$

where $\prod =(C_{11}C_{22}-C_{12}^{2})/C_{66}$, A = sin*θ*, and B = cos*θ*, with *θ* being the angle to the armchair direction.

## 3. Results and Discussion

### 3.1. Structure and Stability

**Figure 1:**
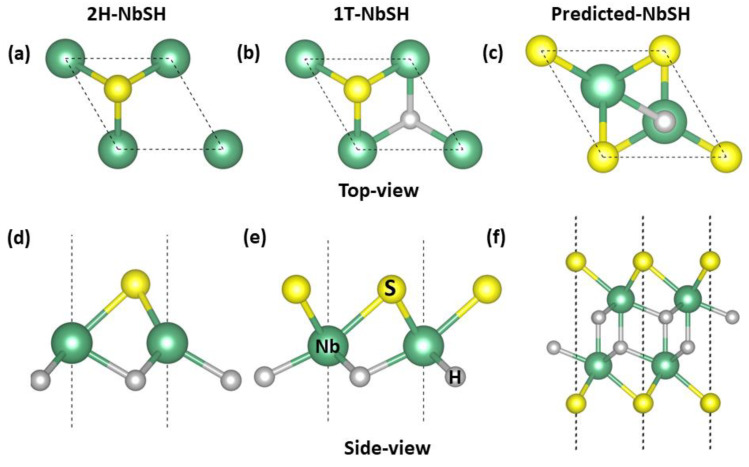
Top view structural diagrams of 2H-NbSH (**a**), 1T-NbSH (**b**), and predicted NbSH (**c**); Side view structural diagrams of 2H-NbSH (**d**), 1T-NbSH (**e**), and predicted NbSH (**f**). Nb, S, and H atoms are represented by green, yellow, and grey spheres, respectively.

Although TMDS has many different phases, such as 2H, 1T, 1T′, 2a × 2a phases, etc., previous work [39] showed that 1T′ and 2a × 2a TMDS phases are not stable phases. Therefore, we mainly examined the 2H- and 1T-NbSH structures in this paper. The top view diagrams of the 2H- and 1T-NbSH are shown in Figure 1a,b, respectively, while their corresponding side views are shown in Figure 1d,e, respectively. Each structure is a layered structure with the space group *P*3*m*1 and includes one unit in the unit cell. The optimized lattice constants of 2H- and 1T-NbSH are 3.1142 Å and 3.1568 Å, respectively. By means of particle-swarm optimization method and density functional theory calculations, the lowest energy structure was predicted, the atomic arrangement of which is illustrated in Figure 1c (top view) and Figure 1f (side view), respectively. The predicted new NbSH phase also has the space group of *P*3*m*1, and it can be viewed as a double layer of 1T-NbSH. The unit cell contains two Nb, two S, and two H atoms in a hexagonal structure with lattice parameters of a and b being 3.2301 Å. It is also worth noting that the out-of-plane mirror symmetry is preserved in this newly found 2D structure.

To examine the stability of NbSH, the cohesive energy per atom in NbSH is defined as $E_{\mathrm{coh}}=(E_{tot}-nE_{\mathrm{Nb}}-nE_{S}-nE_{H})/3n$, where $E_{tot}$, $E_{\mathrm{Nb}}$, $E_{S}$ and $E_{H}$ are the total energies of one unit cell, isolated Nb, S, and H atoms, respectively. The more negative the cohesive energy of a monolayer, the more thermodynamically stable the structure. The calculated cohesive energy of predicted NbSH is −6.72 eV/atom, which is lower than the two known structural values of 2H-NbSH (−6.41 eV/atom), and 1T-NbSH (−6.46 eV/atom). By comparing the cohesive energy of the three phases, we can conclude that the predicted NbSH is more stable thermodynamically than the 2H/1T phases. Additionally, the cohesive energy value of the predicted phase is also lower than that of other typical 2D materials such as MoS_2_ (−4.97 eV/atom) [59], silicene (−3.98 eV/atom), germanene (−3.2 eV/atom) [60], and phosphorene (−3.61 eV/atom) [61,62], demonstrating good thermodynamic stability of the predicted phase.

**Figure 2:**
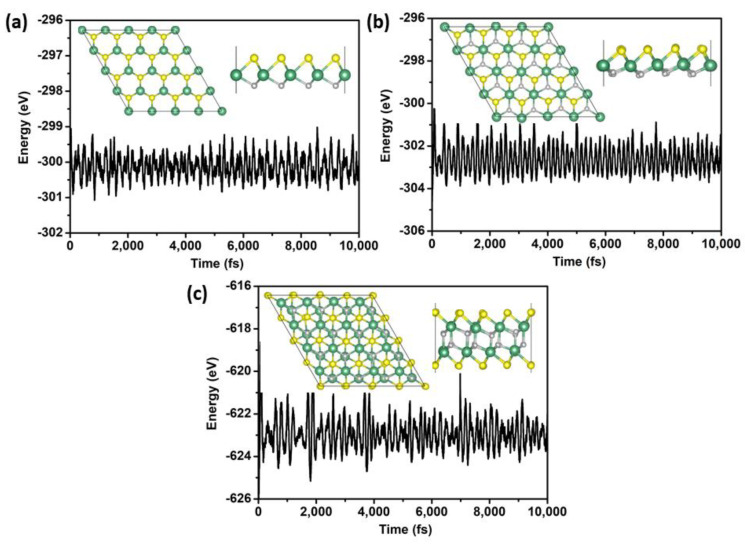
Vibration of total potential energy of 2H−NbSH (**a**), 1T−NbSH (**b**), and predicted NbSH (**c**) during the AIMD at the temperature 300 K. The inset is the final snapshot of NbSH with its top view and side view at the end of 10 ps.

To further evaluate the stability of the three NbSH phases against thermal fluctuations, AIMD simulations were performed employing the NVT ensemble. The final snapshots taken at the end of each simulation are shown in the inset of Figure 2. From Figure 2, it can be seen that each NbSH structure remains intact and no bond is broken. The results indicate that all the NbSH have good thermal stability at a temperature of 300 K. We also calculated the phonon curves of the 1T/2H NbSH, which are shown in Figure S1 in the Supplementary Materials. It was found that there are no negative frequencies in the entire Brillouin zone (BZ), confirming the dynamical and structural stability. As for the predicted NbSH phase, its phonon curve also meets the requirement of dynamical stability, as discussed in more detail later.

**Figure 3:**
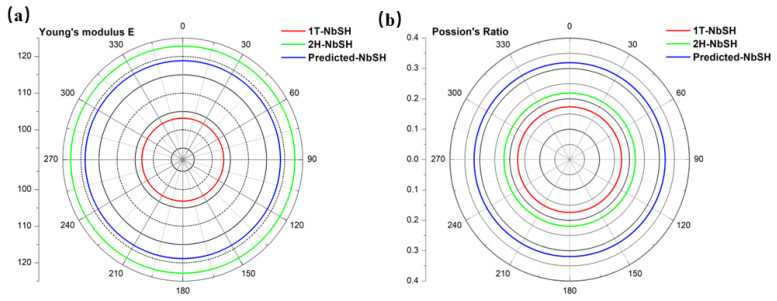
The orientation angle-dependent 2D Young’s modulus (**a**) and Possion’s ratio (**b**) of various NbSH monolayers.

In addition, the elastic constants of NbSH were also evaluated to examine the structural mechanical stability. The second-order elastic constants (SOECs) are crucial in determining the mechanical and dynamical properties of materials, especially their stability and stiffness. The calculated elastic constants are listed in Table 2. For the 2D hexagonal structure, there are two independent elastic constants C_11_ and C_12_, while the non-independent elastic constant is determined as $C_{66}=(C_{11}-C_{12})/2$. It is obvious that all the elastic constants satisfy the necessary mechanical equilibrium conditions $C_{11}C_{22}-C_{12}^{2}>0$ and $C_{11}$, $C_{22}$, $C_{66}>0$ [62], confirming the mechanical stability of all the 2D NbSH. Moreover, the Poisson’s ratio and Young’s modulus as a function of the angle for the three NbSH phases are shown in Figure 3a,b, respectively. It can be seen that all three phases display isotropic mechanical behavior. The in-plane Young’s modulus of NbSH are 122.8, 103.1, 118.8 N/m for 2H-NbSH, 1T-NbSH, and new NbSH phases, respectively. The in-plane Young’s moduli of all three structures are higher than those of silicene (61 N/m), germanene (42 N/m) [59], and black phosphorene (83 N/m) [63], and comparable to that of MoS_2_ (129 N/m) [64]. The Poisson’s ratio characterizes the material’s resultant strain in the longitudinal direction for a material under lateral stress. All the Poisson’s ratios of the three phases also show isotropic behaviors, just like the Young’s moduli. The Poisson’s ratio values of the 2H-NbSH, 1T-NbSH, and new NbSH phases are 0.22, 0.17, and 0.32. The predicted NbSH has the largest Possion’s ratio, indicating a more sensitive structural response to external strain of the predicted structure.

### 3.2. Electronic Properties

**Figure 4:**
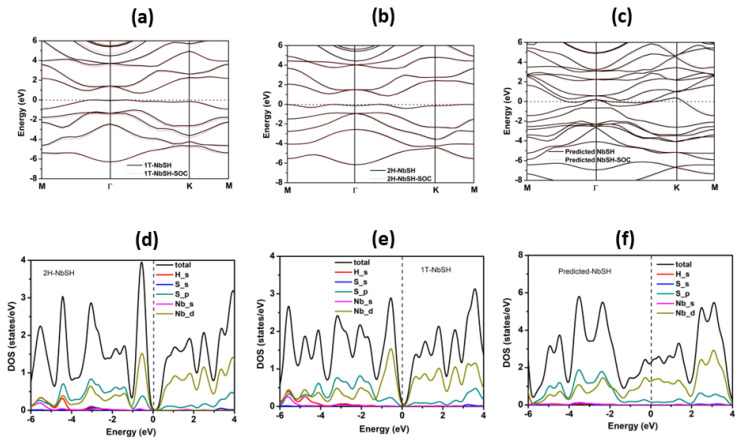
Energy band structures of the NbSH derived from the calculation of DFT-PBE (black lines) and DFT-PBE-SOC (red lines) for (**a**) 2H-NbSH, (**b**) 1T-NbSH, and (**c**) predicted NbSH; the Fermi level (dotted line) is set to zero. The projected density of states of NbSH for (**d**) 2H-structure, (**e**) 1T-structure, and (**f**) predicted structure.

After verifying that NbSH had structural stability, the electronic properties including energy band structures and density of states were examined. As presented in Figure 4a,b the results show that the 2H-NbSH and 1T-NbSH are indirect band gap semiconductors with band gaps of 0.66 and 0.81 eV, respectively. We also calculated the magnetic behavior of the three phases. The corresponding total densities of states (TDOS) with spin-polarized effect considered are shown in Figure S2 in the Supplementary Materials. The upper and lower TDOS are symmetric, indicating that the three phases are non-magnetic. When calculating the band structures of the three phases, we further considered the spin-orbit coupling (SOC) effect. Although the SOC results in some slight spin-slitting in the bands, overall, the SOC effect is not significant, as shown in Figure 4. The total and projected densities of states of the two structures are presented in Figure 4d,e, respectively. The valence band maximum and conduction band bottom of these two structures are composed of d-orbitals of Nb atoms and p-orbitals of S atoms. For the new predicted NbSH structure, the band structure and density of states are shown in Figure 4c,f, respectively. It was found that this structure exhibit metal character and the Fermi level is mainly occupied by the d-orbital of Nb.

### 3.3. Superconductivity

**Figure 5:**
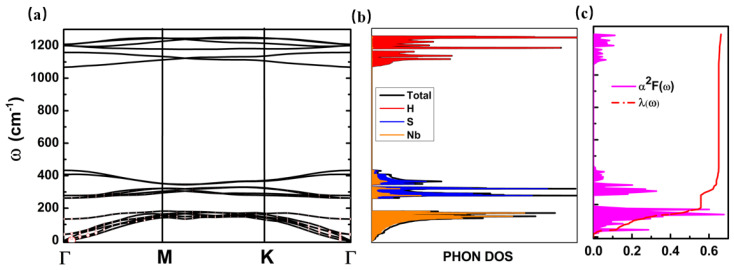
(**a**) Calculated phonon dispersion, (**b**) total and partial phonon density of states, and (**c**) Eliashberg function with integrated EP coupling constant *λ*(ω) for predicted NbSH monolayer. Hollow red circles in (**a**) indicate the phonon linewidth *γ* with a radius proportional to the strength.

Since superconductivity is found in some TMDS and MoSH, we also estimated the superconducting transition temperature $T_{C}$. The λ and $\omega_{\log}$ as functions of ω, together with the phonon dispersion and the projected phonon density of states, are shown in Figure 5. The corresponding λ and $\omega_{\log}$ for the predicted NbSH were found to be 0.71 and 170.7 K, respectively. The corresponding $T_{C}$ of the predicted 2D NbSH is about 6.10 K when setting μ* to 0.1. The electron–electron coulomb repulsion parameter μ* is an empirical constant, the typical value of which is about 0.1–0.3. The different value of μ* can lead to slight deviations of the $T_{C}$ results. In this work, we set μ* to 0.1 based on previous experiences of similar systems, such as bulk NbS_2_ [65] and 2D Janus MoSH [39]. Our predicted 2D NbSH phase exhibits a similar $T_{C}$ value when compared with other reported layered TMDS, such as TaS_2_ with $T_{C}$ below 2.0 K [66], NbS_2_ with a $T_{C}$ of approximately 3 K [67], and WS_2_ with a $T_{C}$ of 8.8 K [68]. However, the $T_{C}$ of our predicted NbSH phase is lower than that of the Janus 1T/2H-MoSH, which has a $T_{C}$ range of about 25–26 K [39]. In addition, from Figure 5a, we can see that there are no negative phonons in the whole BZ zone, indicating the predicted 2D NbSH is also dynamically stable.

We further analyzed the origination of superconductivity of the predicted NbSH. Analysis of the total and partial phonon density of states in Figure 5b suggests that the high-frequency vibrations above 1070 cm are dominated by H atoms, while the intermediate-frequency vibrational region from 250 cm to 436 cm exhibits a strong mixture of S and Nb atoms. The low-frequency vibrations below 190 cm are dominated by Nb atoms. Based on the BCS theory, the electron-phonon coupling constant λ can be integrated into the phonon linewidth $\alpha^{2}F(\omega )$ over the frequency ω using Equation (3). Therefore, the origin of λ can be understood by analyzing $\alpha^{2}F(\omega )$. $\alpha^{2}F(\omega )$ is a physical quantity that depends on the frequency ω of atomic vibrations and the phonon linewidth *γ*, as described by Equation (4). Hence, we can analyze corresponding relationships between *λ* and *ω*/γ. The low-frequency vibrational modes determined by Nb atoms below 190 cm comprised approximately 78.5% of the total *λ*, as illustrated in Figure 5c, indicating that the superconductivity of NbSH mainly originates from Nb atomic vibrations. Furthermore, as shown in Figure 5a, in phonon dispersions, the hollow red circles indicate the phonon linewidth *γ* with a radius proportional to the strength. We can therefore see that *γ* shows large contributions around the Γ point, so that the superconductivity of the predicted phase in this work mainly comes from the Nb atomic vibrations around the Γ point. Contrarywise, for both the 2D Janus 1T/2H MoSH, the superconductivities were reported to originate mainly from the Mo atomic vibrations at the M point [39].

We also applied a biaxial strain to investigate the stress-induced changes in superconductivity, and the calculated results are shown in Table 3. It was found that with increasing strain, the total λ initially increases and then decreases, while $\omega_{\log}$ increases linearly overall. Even a small biaxial strain of −1.08% can increase $T_{C}$ to approximately 9.38 K, suggesting that the superconductivity of the predicted 2D NbSH is very sensitive to biaxial strain.

## 4. Conclusions

In conclusion, we investigated the possible structures of the 2D Janus NbSH monolayer by means of first-principles calculations. We showed that both 2H and 1T-NbSH monolayer meet the stability, mechanical, and dynamical requirements. However, unlike the metallic Janus 2H/1T-MoSH monolayer, both 2H and 1T-NbSH monolayers were unexpectedly found to be semiconductors. We also explored the possible stable NbSH monolayers using an ab initio swarm-intelligence global minimum structure-searching methods. A newly stable 2D MoSH with metallic character was identified for the first time. We showed that the predicted phase is an intrinsic phonon-mediated superconductor, which has a $T_{C}$ of 6.1 K under normal conditions. Interestingly, we found that the $T_{C}$ for the newly found phase is relatively sensitive to applied stress. With a small strain of −1.08, the $T_{C}$ can increase to 9.39 K. We further demonstrated that the superconducting behavior of the newly predicted 2D NbSH is mainly attributed to the Nb atomic vibrations around the Γ point. Our work highlights the structural diversity of TM-SH materials and suggests the possibility of discovering more TM-SH with new physical and chemical properties in the future. In fact, we found that the newly found NbSH phase also appears in other TM-SH 2D materials during our initial structure prediction results. Our investigations provide an essential theoretical basis and an experimental reference for the investigation of more 2D TM-SH materials in the future.

## Figures and Tables

**Table 1:** List of strain modes and the derived elastic constants for the 2D hexagonal system used, based on energy-strain approach.

Strain Index	Strain Vector ε	Elastic Energy $\frac{\Delta E}{V}$
1	(δ, 0, 0)	$\frac{1}{2}C_{11}\delta^{2}$
2	(δ, δ, 0)	${(C}_{11}+C_{12})\delta^{2}$

**Table 2:** Calculated elastic constants of 2H-NbSH, 1T-NbSH, and predicted NbSH.

Structure	*C*_11_	*C*_22_	*C*_12_	*C*_66_
2H-NbSH	129.0	129	28.3	50.4
1T-NbSH	106.3	106.3	18.5	43.9
Predicted NbSH	132.3	122.5	42.2	45.0

**Table 3:** Dependence of the λ, ω_log_, and *T*_C_ on the in-plane biaxial strain of the predicted NbSH.

Biaxial Strain	*λ*	*ω*_log_ (cm^−1^)	*T*_C_ (K)
+0.01%	0.70	163.6	5.40
0.00%	0.71	171.1	6.10
−0.06%	0.80	162.3	7.62
−1.08%	0.81	194.7	9.38
−1.38%	0.73	196.1	7.43
−0.02%	0.64	221.8	6.03

## Data Availability

Data is available from the authors.

## Supplementary Materials

The following supporting information can be downloaded at: https://www.mdpi.com/article/10.3390/molecules28145522/s1, Figure S1: The phonon dispersion relations. (a) 2H-NbSH, (b) 1T-NbSH. Figure S2: The calculated total density of states (TDOS) of the three NbSH phases with spin-polarized effect considered. (a) 1T-NbSH, (b) 2H-NbSH, and (c) predicted-NbSH.

## Sample Availability

Samples of the compounds are available from the authors.
