# Supplementary material for: Structure, Stability, and Superconductivity of Two-Dimensional Janus NbSH Monolayers: A First-Principle Investigation
Source: Molecules. 2023 Jul 19;28(14):5522. doi: 10.3390/molecules28145522 (PMC10385167; doi:10.3390/molecules28145522)

Supplementary Mastherial for

Structure, Stability, and Superconductivity of Two-Dimensional Janus NbSH Monolayers: A First-Principle Investigation

Yan Li [1], Chunying Pu[2,*], and Dawei Zhou[2,*]

[1] Institute for Computational Materials Science, School of Physics and Electronics, International Joint Research Laboratory of New Energy Materials and Devices of Henan Province, Henan University, Kaifeng 475004, China

[2] Henan International Joint Laboratory of MXene Materials Microstructure, College of Physics and Electronic Engineering, Nanyang Normal University, Nanyang 473061, China;

* Correspondence: puchunying126.com(C.P.); zhoudawei@nynu.edu.cn(D.Z.)

Figure S1 The phonon dispersion relations (a) 2H-NbSH (b)1T-NbSH.

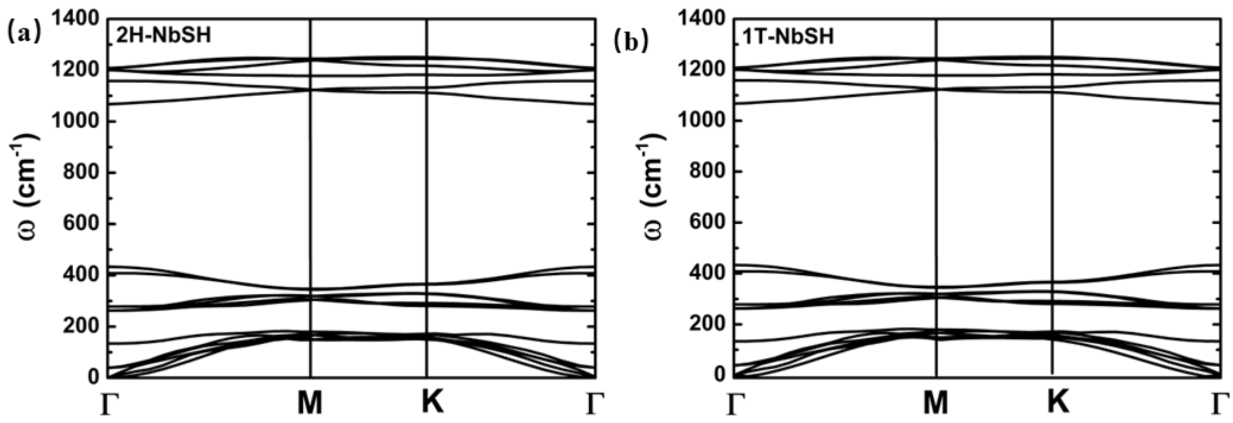

Figure S2 The calculated total density of states (TDOS) of the three NbSH phases with spin-polarized effect considered (a)1T-NbSH, (b) 2H-NbSH and (c)Predicted-NbSH

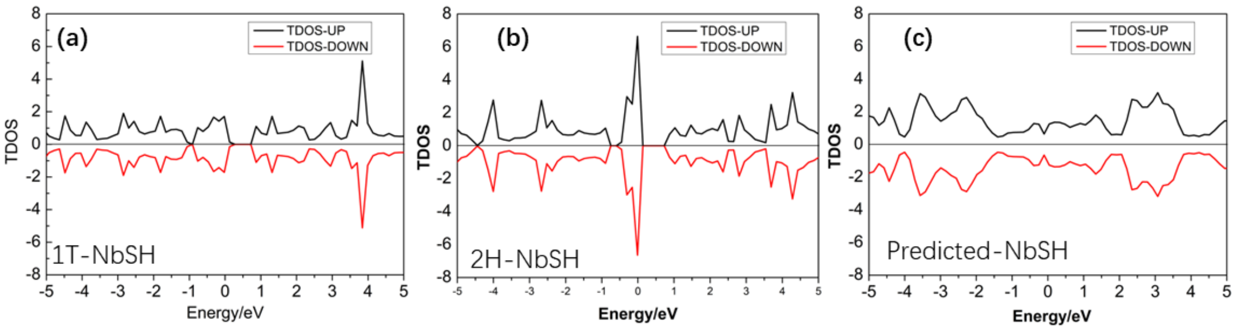

Supplement: Supplementary file 1 [file molecules-28-05522-s001.zip › molecules-2477276-supplementary.pdf]
